# Electroencephalographic Patterns Associated with the Pharmacokinetics and Pharmacodynamics of GABAergic Agents, Opioids, and Ketamine During General Anesthesia: A Systematic Review

**DOI:** 10.3390/jpm16080406

**Published:** 2026-07-29

**Authors:** Alessandro Girombelli, Piergiuseppe Volpe, Francesco Saglietti, Dana Shiffer, Giulia Sette, Raymond M. Planinsic, Francesco Vetrone

**Affiliations:** 1 Department of Emergency Medicine, Unit of Anesthesia and Critical Care Medicine, Ospedale SS. Annunziata, 12038 Savigliano, Italy; alessandro.girombelli@aslcn1.it; 2 Post-Graduate Anesthesia Nursing Program, Higher Specialized School of Nursing (CPSI), Via Cantonale 2C, 6928 Manno, Switzerland; piergiuseppe.volpe@edu.ti.ch; 3 Department of Anesthesiology and Critical Care Medicine, Azienda Ospedaliera S. Croce e Carle, 12100 Cuneo, Italy; 4 Department of Emergency Medicine, IRCCS Humanitas Research Hospital, Rozzano, 20089 Milan, Italy; dana.shiffer@humanitas.it; 5 Department of Biomedical Sciences, Humanitas University, Pieve Emanuele, 20072 Milan, Italy; 6 Department of Systems Medicine, Division of Internal Medicine and Center for Hypertension, University of Rome ‘Tor Vergata’, 00133 Rome, Italy; 7 Department of Anesthesia and Perioperative Medicine, University of Pittsburgh Medical Center, Pittsburgh, PA 15260, USA; planinsicrm@anes.upmc.edu; 8 Department of Anesthesiology, Pain Medicine and Intensive Care Unit, Policlinico San Pietro—Gruppo San Donato, Ponte San Pietro, 24036 Bergamo, Italy

**Keywords:** electroencephalography, general anesthesia, propofol, ketamine, opioid analgesics, remifentanil, pharmacodynamics, pharmacokinetics, depth of anesthesia

## Abstract

**Introduction:** Neuromonitoring during general anesthesia (GA) is recommended to reduce the incidence of postoperative delirium and intraoperative awareness, particularly during total intravenous anesthesia. Guidelines emphasize that anesthesiologists should not rely solely on processed depth-of-anesthesia indices such as the Bispectral Index or Patient State Index but should also interpret the raw electroencephalographic (EEG) waveform and the density spectral array (DSA). Although EEG patterns associated with individual anesthetic agents or combinations of hypnotics and opioids have been described, limited evidence exists regarding EEG activity during multimodal anesthetic regimens. This systematic review aimed to evaluate DSA patterns as pharmacodynamic markers of the cortical effects of GABAergic anesthetics, opioids, and ketamine. **Methods:** PubMed, Embase, and the Cochrane Library were searched without date restrictions up to September 2025. Eligible studies included adult patients undergoing general anesthesia and reporting raw EEG data or specific DSA patterns associated with the investigated drugs. Risk of bias was assessed using RoB 2 and ROBINS-I according to study design. Owing to the limited number of eligible studies, findings were synthesized narratively. **Results:** Out of 273 papers screened, 3 studies met the inclusion criteria, comprising 72 patients. The studies achieved an appropriate and stable effect-site concentration of propofol–remifentanil GA, demonstrated by a baseline DSA recorded before ketamine administration. Ketamine administration produced a shift from the baseline alpha–delta pattern to a beta–delta DSA pattern. **Conclusions:** Ketamine administration during stable propofol–remifentanil anesthesia produces a characteristic shift towards a beta–delta DSA pattern, which may increase processed EEG indices, leading to misinterpretation of anesthetic depth. Further studies are needed to characterize DSA signatures associated with multimodal anesthesia and to identify patterns indicative of adequate anesthetic depth when multiple agents are administered.

## 1. Introduction

Depth of anesthesia (DOA) monitoring during general anesthesia (GA) is currently recommended by the European Society of Anaesthesiology and Intensive Care (ESAIC) to reduce the risk of intraoperative awareness and postoperative delirium, as it allows for a more tailored anesthetic management strategy. Current guidelines emphasize that anesthesiologists must be able to interpret raw electroencephalography (EEG) signals and density spectral array (DSA) patterns, as processed indices alone are insufficient for optimal management of patients under GA [1]. DSA represents a time–frequency representation of EEG power across different frequency bands, allowing real-time visualization of anesthetic-induced cortical oscillations. Specific DSA patterns have been described for various anesthetic agents [1,2]. However, evidence describing EEG activity during multimodal anesthesia remains limited. Importantly, EEG features vary substantially according to patient age and neurocognitive reserve [3], reinforcing the need for individualized interpretation of EEG and DSA patterns.

Volatile anesthetics have historically been titrated using the Minimum Alveolar Concentration (MAC), defined as the anesthetic’s alveolar concentration at which 50% of patients do not respond to a standardized surgical stimulus [4]. Although MAC correlates with the partial pressure of anesthetic agents in the central nervous system, it remains an empirical measure derived from healthy volunteers [4] and based primarily on behavioral responses rather than neurophysiological markers. Moreover, MAC is influenced by multiple factors such as age, body temperature, comorbidities, and the concomitant use of opioids or sedatives. Consequently, relying solely on MAC may not accurately reflect the true hypnotic state of the brain and may lead to excessive sedation or even burst suppression, particularly in elderly or vulnerable patients, since the alveolar concentration needed to induce unconsciousness may differ from the MAC value [3]. DOA monitoring therefore allows clinicians to tailor volatile anesthetic dosing according to each patient’s neurophysiological response [5], thus improving patient safety.

Intravenous anesthetics such as propofol and remifentanil are increasingly delivered using target-controlled infusion (TCI) systems based on pharmacokinetic/pharmacodynamic (PK/PD) models [6,7]. These systems aim to achieve specific plasma or effect-site concentrations in order to provide adequate hypnosis or analgesia while minimizing adverse effects associated with drug accumulation or excessive dosing, such as hypotension or burst suppression. Early models, derived from relatively small cohorts of healthy volunteers [6,8], linked plasma concentrations to clinical effects and were later updated to incorporate effect-site targeting based on processed DOA indices [9]. However, these models often failed to account for interindividual variability, particularly in specific populations such as obese patients or children, as they relied primarily on compartmental analysis and assumed linear pharmacokinetics across all patients [10].

More recently, population-based nonlinear mixed-effects models (NONMEM) have been developed to better account for interpatient variability and improve predictive accuracy [7,11]. The integration of pharmacological modeling and neurophysiological monitoring has significantly advanced the understanding and control of anesthetic depth [12]. When combined with EEG and DSA monitoring, TCI systems enable real-time correlation between predicted drug concentrations and brain electrical activity, thereby supporting individualized drug titration and reducing the risks of hypotension, awareness, and burst suppression.

The integration of DSA with conventional processed EEG indices has allowed researchers to link effect-site drug concentrations with characteristic DSA signatures. At adequate concentrations, propofol produces a frontal alpha–delta pattern, characterized by alpha oscillations (8–12 Hz) superimposed on a slow delta background (0.5–4 Hz) [2,13].

In contrast, volatile anesthetics (i.e., sevoflurane, isoflurane, etc.) typically induce a theta–delta pattern, reflecting a broader cortical depression effect [2,4]. This slower frontal background rhythm may reflect differences in PD mechanisms or anesthetic potency compared with propofol [14]. Additionally, because MAC values were historically established based on clinical responsiveness rather than neurophysiological monitoring, anesthetic concentrations considered clinically adequate may, in some cases, correspond to deeper levels of cortical suppression [15].

**Figure 1:**
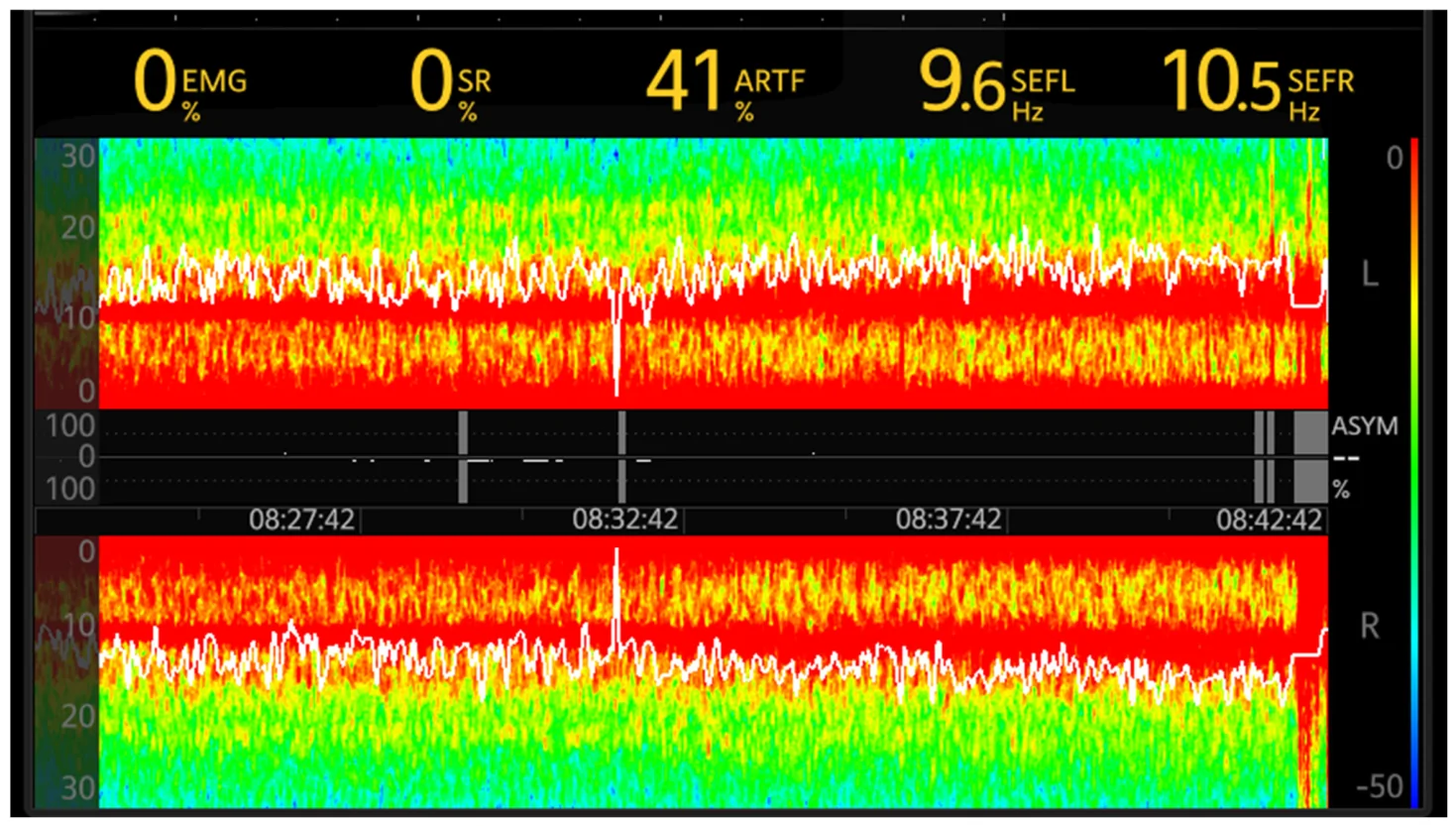
A Density Spectral Array (DSA) pattern of propofol and remifentanil.

**Figure 2:**
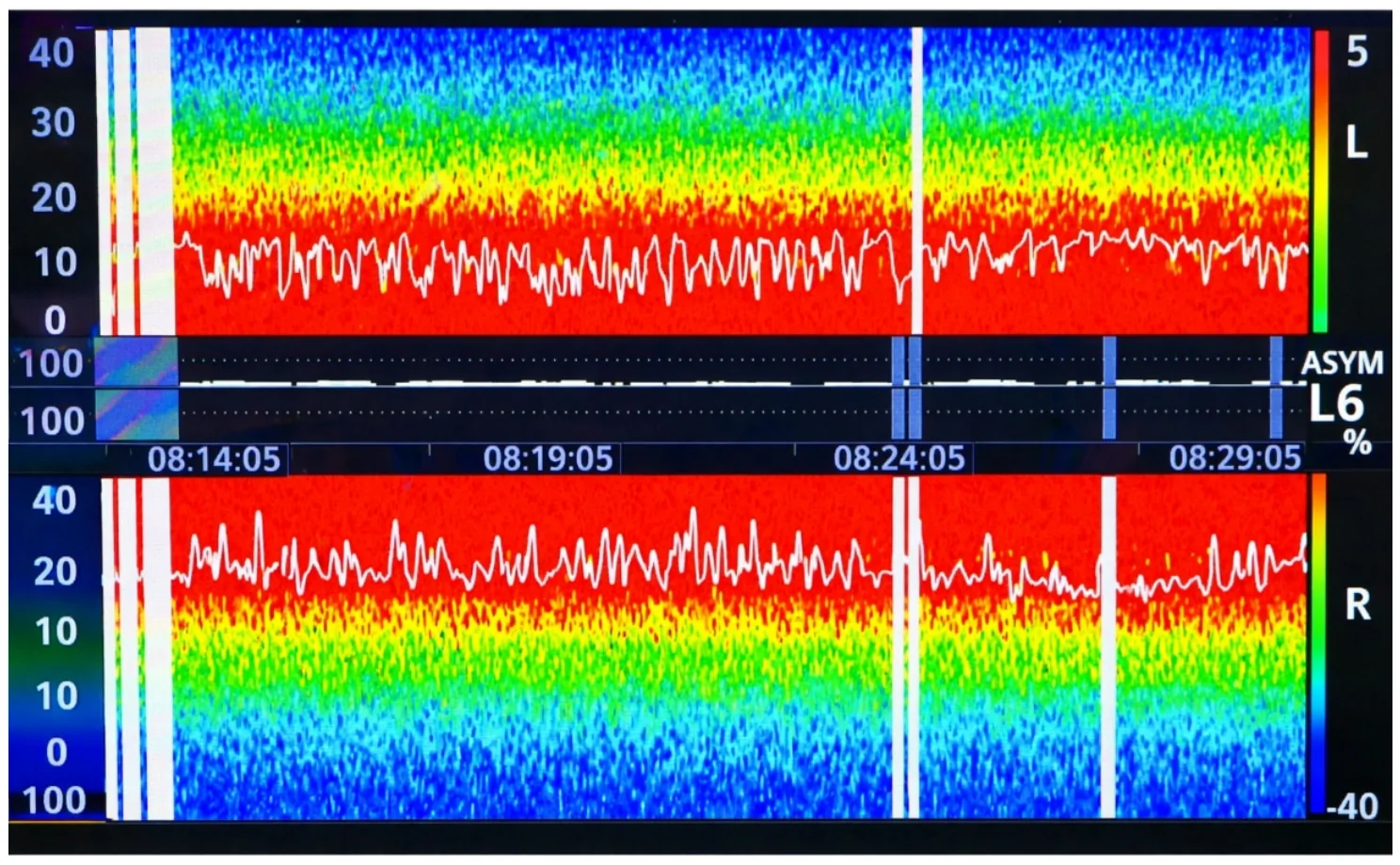
A Density Spectral Array (DSA) pattern of sevoflurane and remifentanil.

Evidence regarding opioid-related EEG signatures remains inconsistent. Some authors have suggested that increases in theta power (4–7 Hz) may represent a marker of adequate analgesia [16]. When combined with GABAergic anesthetics, opioids may contribute to the maintenance of stable alpha–delta rhythms by reducing nociception-driven cortical arousal (i.e., preventing abrupt shifts towards beta–delta patterns) [17] (Figure 1 and Figure 2).

**Figure 3:**
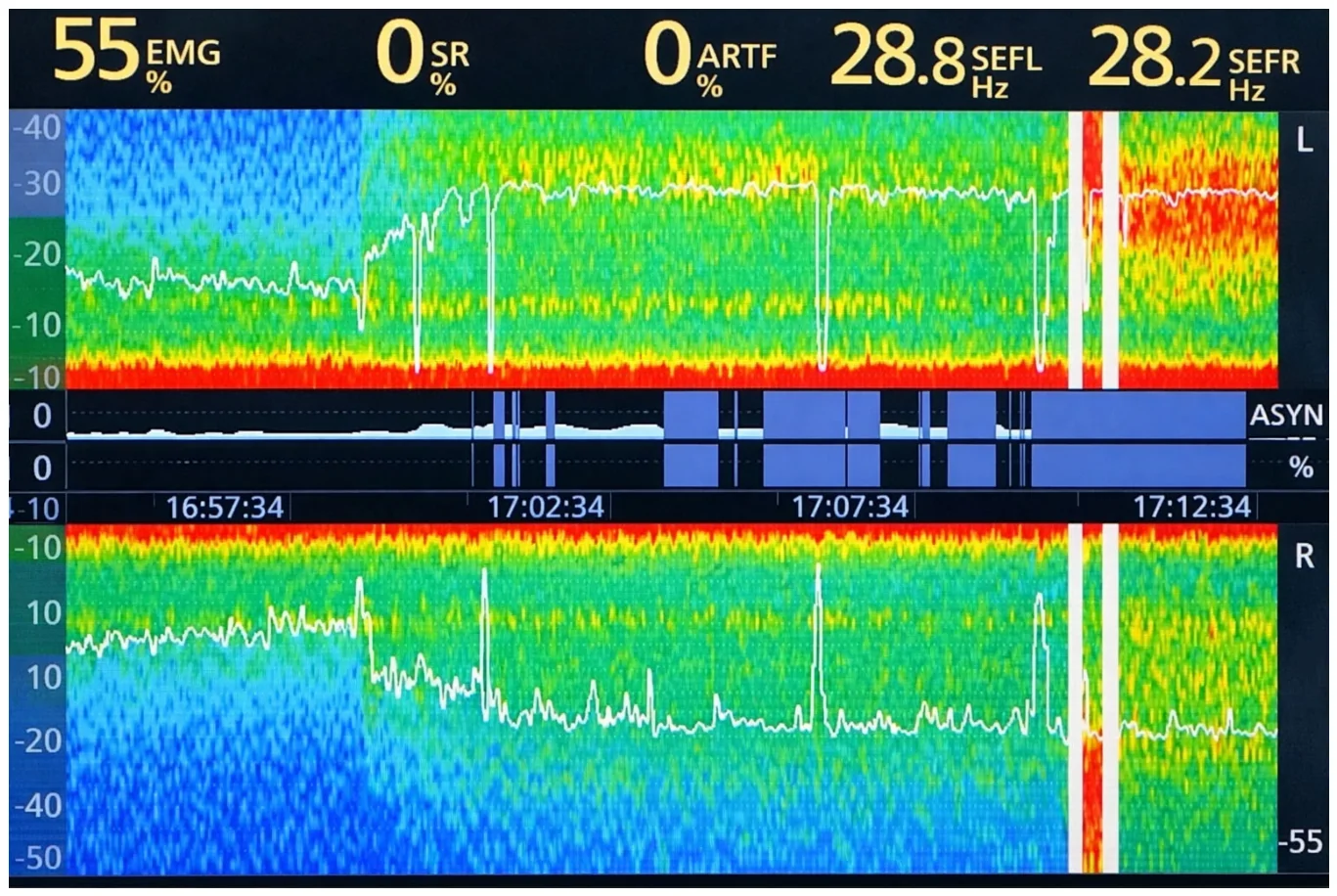
A Density Spectral Array (DSA) pattern of 1 mg/kg bolus dose of ketamine followed by sevoflurane 0.6 MAC.

N-methyl-D-aspartate (NMDA) receptor antagonists, particularly ketamine, produce distinct electrophysiological signatures. When administered at induction doses (i.e., 1–2 mg/kg iv), ketamine typically produces a gamma burst pattern (>30 Hz) superimposed on a mixed-frequency background. In contrast, subanesthetic doses (i.e., <1 mg/kg) or the waning phase following an induction dose are associated with a low beta–delta pattern [18] (Figure 3).

Most available studies have focused on characterizing DSA patterns associated with single agents or combinations of GABAergic agents and opioids [1,2]. However, contemporary anesthetic practice increasingly relies on multimodal approaches combining hypnotics, analgesics, NMDA-antagonists, and other adjuncts [19]. In this context, understanding how multiple drug classes interact pharmacodynamically and how these interactions manifest on DSA becomes increasingly important. Real-time visualization of these combined electrophysiological effects may help guide drug titration to optimize hypnosis, analgesia, and cortical stability, particularly in elderly or neurologically vulnerable patients. Once this step is completed, subsequent studies should focus on determining whether specific DSA alterations, such as a reduction in band-specific power or a shift in dominant oscillatory frequencies, correlate with clinically relevant outcomes, including an increased risk of delirium or mortality.

This review therefore aims to investigate the DSA patterns associated with the combined administration of GABAergic anesthetics, opioids, and ketamine during general anesthesia, given the increasing use of multimodal anesthetic techniques and the current lack of detailed characterization of their combined electrophysiological effects.

## 2. Materials and Methods

A systematic review was conducted according to the Preferred Reporting Items for Systematic Reviews and Meta-Analyses (PRISMA) 2020 statement (Supplementary Table S2) [20]. The review aimed to identify studies evaluating EEG and DSA patterns associated with the combined use of GABAergic anesthetics, opioids, and ketamine during general anesthesia. The review was prospectively registered in the International Prospective Register of Systematic Reviews (PROSPERO; CRD420251175673), where the review protocol is publicly available.

The primary objective of this review was to analyze specific DSA patterns associated with the combined administration of GABAergic anesthetics, opioids, and ketamine.

A comprehensive literature search was performed in Embase, MEDLINE, and the Cochrane Library from inception to September 2025, without date restrictions. The same combination of keywords was used across the three databases. The complete search strategy is provided in Supplementary File S1.

The inclusion criteria were adult patients (>18 years), retrospective studies or prospective clinical trials conducted under general anesthesia, studies reporting raw EEG data or specific DSA patterns associated with the studied drugs, and articles published in English.

The exclusion criteria were pediatric populations, case series or case reports, studies reporting only numerical EEG indices without DSA patterns, studies involving light sedation, non-human studies, and non-English language articles.

The screening process of the articles was performed independently by two authors applying the predefined inclusion and exclusion criteria.

Any discrepancies between reviewers were resolved by consultation with a third author who was blinded to the initial decision. All potentially eligible articles underwent full-text assessment to confirm inclusion. Data extraction was independently conducted by two authors, with disagreements resolved through the process mentioned above. The relevant data from each included study are summarized in Table 1.

Risk of bias was assessed using the appropriate tool based on the study design: the Cochrane Risk of Bias 2 (RoB 2) tool for randomized controlled trials and the Risk Of Bias In Non-randomized Studies of Interventions (ROBINS-I) tool for non-randomized studies. The certainty of evidence was evaluated using the Grading of Recommendations Assessment, Development and Evaluation (GRADE) framework. Due to the scarcity of eligible studies, quantitative analysis was not feasible. Therefore, the results are presented narratively.

## 3. Results

**Figure 4:**
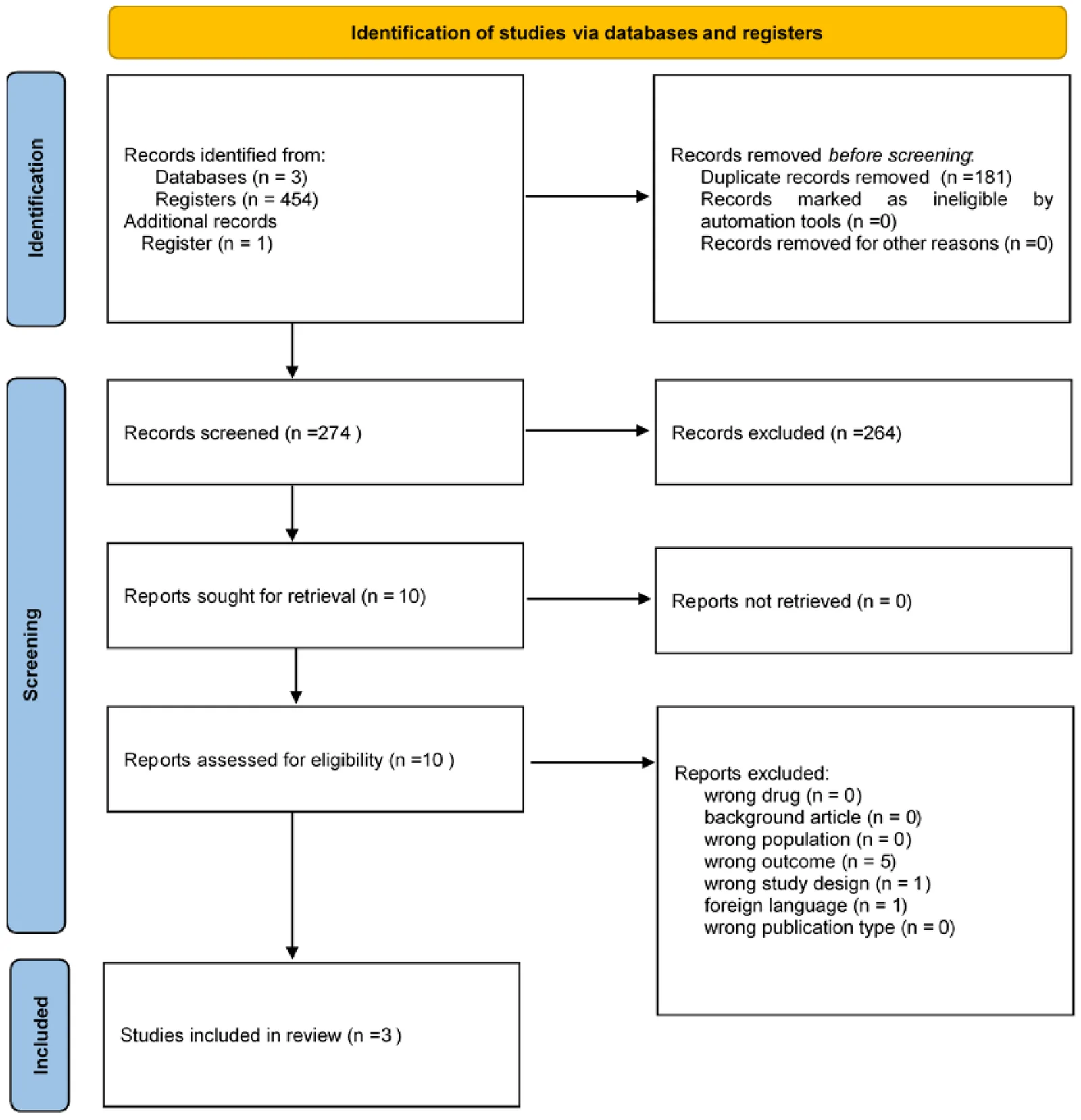
PRISMA 2020 flow diagram [20].

A total of 454 articles were identified through the search strategy. After the removal of 181 duplicate records, 273 articles were screened based on abstract information. Of these, 264 were excluded according to the inclusion criteria described in the Methods section. Nine articles underwent full-text review, of which seven did not meet the inclusion criteria (Details in Supplementary Table S3). One article was published shortly after the time frame (i.e., September 15th, time frame ended on September 1st) was added due to its clinical relevance. Ultimately, three articles were included in the final analysis. The results are summarized in Figure 4. The relevant data from each included study are summarized in Table 1.

Linassi et al. [21] conducted a single-center, prospective, observational trial including 14 adult female patients classified as ASA I who underwent oncologic breast surgery between March 2022 and January 2023. The primary outcome was the dose-dependent effect of ketamine on the Bispectral Index (BIS), Spectral Edge Frequency (SEF), and the Surgical Pleth Index (SPI), a marker of intraoperative nociception, during stable propofol and remifentanil target-controlled infusion total intravenous anesthesia (TCI-TIVA). Propofol was administered using the Eleveld TCI model with an effect-site concentration of 2–3 μg/mL in patients >50 years and 3–4 μg/mL in patients <50 years. Remifentanil was administered using the Minto model with effect-site concentrations of 2 ng/mL (>50 years) or 3 ng/mL (<50 years). Adequate anesthesia was assessed by loss of consciousness (LOC) and achieving the desired infusion concentration, while BIS monitoring (range 40–60) was used during maintenance. Ketamine was administered using the Domino TCI model. The infusion was started at 1 μg/mL, and once this target concentration had been reached, it was subsequently reduced to 0 μg/mL. Although DSA was not included as a predefined outcome, it was calculated based on their collected data, allowing a qualitative description of the spectral changes observed. Key findings included increases in both BIS and SEF values, with no significant changes in SPI. Notably, during propofol–remifentanil anesthesia, the authors described a characteristic increase in alpha–delta band oscillatory patterns. Following ketamine administration, low beta oscillatory activity appeared, which gradually returned to an alpha pattern as ketamine plasma concentration decreased. The risk of bias for this study was assessed using the ROBINS-I tool and is summarized in Table 2.

The overall risk of bias was classified as serious, mainly due to the absence of a control group and the lack of covariate adjustment or mixed-effects modeling, which the authors reported was not feasible due to a small sample size. Potential residual confounders included age, sex (all participants were female), and nociceptive stimuli during surgery. Furthermore, the high exclusion rate (78%) introduced significant selection bias, as the cohort represents a highly selected population of pharmacodynamic responders, thus limiting the generalizability of the findings. According to the GRADE framework, the overall certainty of evidence was considered very low.

Araujo et al. [22] conducted a randomized controlled trial (RCT) including 39 adult patients undergoing elective spine surgery. General anesthesia was induced and maintained using a TCI-TIVA protocol for both propofol and remifentanil. After stable anesthesia was achieved, patients were randomized to receive a ketamine bolus of 0.1 mg/kg (group 1), 0.5 mg/kg (group 2), or 1 mg/kg (group 3). Following ketamine administration, SEF increased by 1.34 ± 0.99 in group 1 (*p* < 0.001). In group 2, SEF increased by 3.03 ± 2.04 (*p* < 0.01) and BIS by 6.65 ± 7.40 (*p* = 0.008). In group 3, SEF and BIS increased by 3.30 ± 2.77 (*p* = 0.01) and 6.85 ± 10.35 (*p* = 0.034), respectively. Additionally, an increase in higher frequency components on the DSA compared with baseline was reported, although neither the baseline DSA pattern nor the specific EEG frequency bands showing increased power were described. The risk of bias was assessed using the RoB 2 assessment tool, with the results summarized in Table 3.

The resulting overall risk of bias was classified as “some concerns”, primarily because no surgical stimulus was present during the observation period. The GRADE certainty of evidence was rated very low. Although the observed effect appears to be dose-related, its magnitude and clinical relevance remain unclear.

Overall, both studies reported an increase in higher-than-alpha frequencies following ketamine administration during propofol–remifentanil anesthesia, consistent with the observed increases in BIS and SEF values.

Tanaka et al. [23] conducted a prospective, single-arm observational study including 19 ASA 1–2 female patients scheduled for elective surgery. Anesthesia was induced with propofol administered with a TCI between 3 and 5 mcg/mL and maintained between 2 and 3.2 mcg/mL; the specific TCI protocol was not specified. Remifentanil was coadministered as a continuous infusion between 0.07 and 0.33 mcg/kg^−1^/min^−1^. Rocuronium was administered to facilitate intubation. After 10 min of stable infusion, ketamine was administered as a 0.5 mg/kg^−1^/bolus, followed by a continuous infusion at 0.125 mg/kg^−1^/min^−1^. Propofol was titrated during the operation to maintain a BIS value of 40–60 before ketamine administration; after ketamine administration, adjustments to the anaesthetics were made based on the clinical judgement of the attending anaesthesiologists, not on BIS values. Patients with modified propofol infusion rates during the study were excluded to isolate the effect of ketamine on PAC. EEG data was acquired using the BIS monitor, and the DSA was obtained with multitaper spectral analysis implemented with Mathlab’s pmtm function. Phase-amplitude coupling analysis was also performed but is beyond the scope of the present paper. The results showed an increase in BIS and SEF95 after ketamine administration. The DSA showed a decrease in alpha and delta power with an increase in beta power after ketamine administration.

The strengths of this study are the use of NMBA to limit muscle interference on the EEG signal; propofol was titrated to a BIS of 40–60 before the administration of ketamine to limit a possible confounder derived by varying propofol doses; and after ketamine administration, the anesthesiologist did not modify the propofol infusion solely based on the BIS value, thus avoiding a critical mistake when coadministering ketamine with GABAergic anesthetics.

The limitations of this study are the lack of formal sample size calculations, instead using a convenience sample size based on prior similar work, which increases the risk of underpowering. All patients were female by institutional practice (TIVA preference in young women for PONV reduction), which limits generalizability. The absence of a control arm not receiving ketamine (e.g., propofol-only comparator) is the largest structural limitation, precluding differentiation of ketamine-specific effects from time-dependent surgical/anaesthetic drift. Propofol was administered with “effector site” targeting, but no mention of the specific protocol is made, and no specific information is provided on whether the protocol was the same in all patients, thus resulting in considerable variation in propofol dosage. Remifentanil was not administered with a TCI protocol, and no explanation was given for this choice; the dosage range was quite wide and encompassed the entirety of the procedure, with no recorded dose for induction, intubation, and surgical incision. Because remifentanil can alter the DSA, the authors should have recorded the dosage of remifentanil being administered during the DSA recording and the time elapsed between the last dose modification and the recording, as this could be a critical confounder.

The risk of bias analysis for this paper was conducted with the ROBINS-I tool (Table 4) and was overall moderate, primarily due to confounding from concomitant drugs/interventions and lack of a fixed post-ketamine titration protocol.

## 4. Discussion

DOA monitoring is commonly performed using specialized devices that process raw EEG signals through proprietary algorithms to generate a dimensionless numerical index. Manufacturers typically provide recommended index ranges for either light sedation or general anesthesia [24]. Most society guidelines endorse monitoring DOA using these recommended index ranges, and the majority of published articles follow those indications. However, in recent years, a more in-depth approach has been proposed involving qualitative analysis of drug-associated DSA patterns, which represent a more informative processed EEG parameter. By evaluating the prevalence and intensity of specific EEG frequencies and patterns, clinicians can better assess the adequacy of anesthesia [2,13]. A major limitation of processed indices arises during ketamine administration. Ketamine increases beta-wave activity when administered during stable GABAergic anesthesia, which elevates the processed index and falsely suggests a lighter depth of anesthesia. This phenomenon can render processed indices unreliable for clinical monitoring [11,25]. In contrast, correct interpretation of the DSA can overcome this limitation by revealing a visible characteristic shift from the stable alpha–delta pattern associated with GABAergic anesthesia to a higher beta–delta pattern following ketamine administration. As ketamine’s effect wanes, the DSA gradually returns to the baseline GABAergic pattern [18,26], closely reflecting the drug’s pharmacokinetic profile. Recently, the ESAIC issued a guideline on perioperative delirium prevention, emphasizing that reliance on processed indices alone is insufficient for accurate monitoring of anesthetic depth [1]. These guidelines highlight the importance of anesthesiologists being able to interpret raw EEG signals and drug-specific DSA patterns in order to reduce the risk of burst suppression from over-sedation and, consequently, postoperative delirium. However, most drug-specific DSA patterns described in the literature involve either single hypnotic agents or combinations of two drugs administered simultaneously [27]. Such studies do not fully reflect the growing multimodal anesthesia practice, which relies on the simultaneous administration of several drugs (i.e., opioids, ketamine, alpha-2 agonists, Magnesium sulfate, etc.), which may substantially alter DSA patterns [19].

Linassi et al. [21] conducted a prospective observational study aimed at investigating the effects of adding a ketamine bolus to stable propofol–remifentanil general anesthesia. Propofol was administered using the Eleveld model, currently one of the most comprehensive TCI models available for propofol. However, the authors did not specify whether they used the version of the model that accounts for the simultaneous administration of remifentanil. Propofol and remifentanil are known to act synergistically [28], and the Eleveld model was specifically developed to account for this interaction. Despite this, the authors chose to administer remifentanil using the Minto model instead.

The Eleveld pharmacokinetic model for remifentanil offers several advantages over the classical Minto model. The Minto model, although extensively validated and still widely used, was developed within a more limited domain and relies on lean body mass (LBM) as a key covariate; this can lead to less reliable pharmacokinetic predictions at extremes of body size or demographics because the underlying James LBM equation can behave anomalously in overweight/obese patients and outside the original development range [29] and is explicitly highlighted as a practical limitation for remifentanil TCI implementations [30]. Consistent with this, TIVA practice guidance notes that Minto’s LBM calculation is only valid within BMI limits (and thus should not be extrapolated indiscriminately to higher BMI ranges) [31].

The Eleveld remifentanil model, by contrast, was developed using an allometrically scaled PK/PD framework designed to be accurate across a wide range of ages and weights, incorporating contemporary covariate modeling (including size scaling and age-related functions), and in its development paper its predictive performance was reported to exceed the Minto model over a wide age and weight range [32]. From a TCI implementation perspective, simulations across children, adults, older people, and individuals with severe obesity further support more consistent dosing behavior with Eleveld-type modeling compared with older structures that are sensitive to LBM pathologies, and they explicitly frame the LBM anomaly in Minto as a reason for concern in obesity [30]. This “general-purpose model” rationale is consistent with contemporary reviews describing the motivation for broad-application PK/PD models (including Eleveld remifentanil) to reduce invalid model selection and improve applicability across neonates/children, obese, and elderly patients [33].

An additional advantage is the internal consistency achieved when both propofol and remifentanil are modeled using the Eleveld framework: the Eleveld propofol model was explicitly developed for broad application using modern covariate structures (allometry and maturation concepts) and avoids problematic LBM formulations [34], aligning naturally with the Eleveld remifentanil model [32]. In practical combined-drug use, simultaneous administration studies have shown no evident PK prediction bias for the Eleveld propofol and Eleveld remifentanil models when used together, supporting compatibility of the framework in coadministration settings [35]. Recent clinical guidance also explicitly considers the Eleveld PK model appropriate for obese patients (including very high body weights), supporting the clinical acceptability of Eleveld-based TCI implementation in heterogeneous populations [36].

Finally, it is worth noting that “more general-purpose/robust” does not imply Minto is unusable: Minto can perform acceptably in certain complex contexts (e.g., normothermic cardiopulmonary bypass) [37,38], but its covariate behavior and original domain restrictions make Eleveld’s broader-population design a more coherent default choice when aiming for one remifentanil model across diverse ages and body sizes [30,32].

Furthermore, ketamine was administered using the Domino model with an effect-site target. This is probably a typographical error, since the Domino model provides only plasma target concentrations, and currently, no model provides an effect-site target concentration for ketamine. The absence of an effect-site model may reflect the fact that a clear DSA pattern corresponding to ketamine effect-site concentrations had been described primarily for anesthetic doses, whereas subanesthetic doses typically produce a beta–delta pattern that does not allow reliable differentiation between dissociated and aware state [26,39].

The results of the study showed that administration of a TCI bolus of ketamine during stable propofol–remifentanil general anesthesia resulted in a DSA shift from an alpha–delta to a beta–delta pattern, while no apparent DSA changes were attributable to the remifentanil infusion. To our knowledge, this represents the first prospective study to investigate the specific DSA patterns associated with the combined administration of these three drugs in a clinical setting. As such, it should be considered an initial exploratory study that may serve as the basis for future investigations focusing specifically on DSA patterns as a primary endpoint.

The main strength of the study lies in the administration of all three agents using their respective TCI pharmacokinetic models, which helps limit variability associated with drug accumulation and overshoot of target plasma concentrations. Nevertheless, several methodological limitations weaken the strength of its conclusions. First, DSA analysis was designated as a secondary rather than a primary outcome, despite increasing recommendations emphasizing its clinical relevance. Second, the exclusion of neuromuscular blocking agents limits the generalizability of the findings, as these agents are commonly used in general anesthesia and improve EEG signal quality by cancelling muscle artifacts; however, the BIS system has a built-in algorithm that identifies high-band-muscle frequencies (20–100 Hz) [40] and categorizes them as a muscle artifact when compiling the DSA. This limits the contamination of the final DSA pattern, albeit with a chance of excluding part of the beta band from the analysis. Third, the combination of the Eleveld TCI model for propofol and the Minto model for remifentanil was not clearly justified and lacks a strong clinical rationale, given that the Eleveld model was specifically designed to account for the coadministration of remifentanil. Fourth, propofol induction dose was based primarily on patient age, and loss of responsiveness was assessed clinically rather than through DSA-guided titration to achieve a characteristic alpha–delta pattern confirming adequate DOA. Finally, the sample size of the study was relatively small and limited to a single type of surgery, further limiting the generalizability of the results.

Araujo et al. [22] conducted a RCT involving patients undergoing elective spine surgery. Anesthesia was induced with propofol administered via TCI at a rate of 200 mL/h until LOC, while remifentanil was delivered using TCI with an effect-site concentration of 3 ng/mL. Once a stable BIS value was achieved, patients were randomized to receive a ketamine bolus of 0.1, 0.5, or 1 mg/kg. The primary outcomes were changes in BIS, SEF, and DSA. The results indicated a significant increase in both BIS and SEF values compared with baseline, accompanied by increases in higher frequency components on the DSA. However, several methodological limitations were noted. Specifically, the baseline DSA pattern was not reported, and the specific EEG frequency bands that increased following ketamine administration were not described. Additionally, the authors did not specify the TCI models used for propofol and remifentanil, nor did they report the selected target effect-site concentration for propofol. Finally, stable anesthesia was assessed using BIS values rather than DSA patterns, despite current international recommendation guidelines [1]. These limitations introduce substantial bias and require that the findings be interpreted with caution.

Tanaka et al. [23] conducted a prospective, single-arm observational study including 20 ASA 1–2 female patients scheduled for elective surgery. Propofol was administered with a non-specified TCI algorithm set to the effector site throughout the procedure. Remifentanil was coadministered as a continuous infusion with a wide dosing range, with no specific mention of the mean dose administered during crucial points in the study (i.e., induction, intubation, incision, during ketamine bolus and continuous infusion). Rocuronium was administered for intubation, and, thus, EEG artifacts resulting from muscle interference were excluded. The results showed an increase in BIS and SEF95 after ketamine administration. The DSA showed a decrease in alpha and delta power with an increase in beta power after ketamine administration. Ketamine was administered after stable anesthesia and, from that point on, the anesthesiologist was not required to maintain BIS between 40 and 60, avoiding a critical pitfall that would have resulted in an erroneous increase in propofol dose; instead, the anesthesiologist used clinical judgment to titrate anesthesia. Data from this time point that reported propofol and remifentanil doses could have been useful to prove adequate anesthesia and analgesia. The same dosage data would have been useful during ketamine bolus and infusion since it would have allowed correlating the dosage of these three drugs with the resulting DSA pattern. This study shows how subanesthetic doses of ketamine result in an increase in beta band power, even in the presence of remifentanil, thus hinting at the hypothesis that the shifting DSA pattern, from an alpha–delta prevalent band power to a low-power alpha and delta with increased beta power could be a useful marker of adequate multimodal analgesia and anesthesia.

Overall, the results of the present systematic review suggest that DSA patterns may allow anesthesiologists to properly monitor DOA, even in the presence of ketamine bolus administration. Adequate DOA may be inferred by recognizing the shift from an alpha–delta pattern to a beta–delta pattern corresponding to the peak pharmacodynamic effect of ketamine, regardless of the processed indices. The subsequent return to the baseline DSA likely reflects declining ketamine concentrations.

Interestingly, this review highlights the scarcity of studies relying solely on DSA interpretation as a marker of DOA, despite the growing body of literature [30] and recent ESAIC guidelines suggesting that processed indices alone are insufficient for accurate anesthetic depth monitoring. This discrepancy likely reflects the typical delay between the publication of updated best-practice guidelines and their actual implementation in clinical practice, particularly given that anesthesiologists are required to learn how to interpret EEG spectrograms and raw EEG signals.

Future research should aim to validate these EEG and DSA patterns in larger and more diverse cohorts, including older and more medically complex patients undergoing a broader range of surgical procedures, in order to enhance the clinical applicability of the findings. Additionally, exploring the different effects of ketamine administered as a single bolus versus continuous infusion may reveal distinct DSA patterns, as continuous infusions could exhibit context-sensitive pharmacokinetics that are not observed with bolus dosing. Further studies should also investigate different TCI models and combinations of effect-site concentrations in order to identify optimal strategies for achieving the desired DOA and DSA patterns while minimizing adverse effects. Incorporating these insights into anesthetic depth monitoring algorithms could improve their accuracy, reduce misinterpretation of cortical arousal, and ultimately enhance patient safety during anesthesia. Nevertheless, several barriers remain to the widespread implementation of these novel approaches and monitoring devices. The demands of routine clinical practice and the specialized nature of neuromonitoring currently hinder their rapid adoption. For this reason, anesthesia residency programs and continuing medical education programs should incorporate dedicated lectures and hands-on training focused on EEG-based DOA monitoring. Such educational efforts would help anesthesiologists remain aligned with rapidly evolving practice guidelines and emerging neurophysiological markers of anesthetic depth.

## 5. Conclusions

In conclusion, the present review suggests that the addition of ketamine, both at subanesthetic and anesthetic doses, to stable propofol–remifentanil anesthesia produces a shift in DSA patterns from an alpha–delta pattern to a beta–delta pattern, corresponding to ketamine’s peak effective concentration. These findings indicate that future studies should focus on DSA pattern analysis as a primary outcome rather than relying solely on processed EEG indices. Furthermore, the DSA signatures associated with different combinations of anesthetic and analgesic agents should be further investigated, particularly as multimodal and opioid-sparing anesthetic strategies become increasingly common and may complicate the interpretation of conventional DOA monitoring.

## Figures and Tables

**Table 1:** Summary of the included trials. Values are reported as mean ± SD or median (interquartile range) when available. TCI: target-controlled infusion; CeP: propofol effect-site concentration; CeR: remifentanil effect-site concentration; BIS: Bispectral Index; SEF: Spectral Edge Frequency; SPI: Surgical Pleth Index; DSA: density spectral array.

Study	Design	Population	Anesthesia Protocol	Ketamine Protocol	Outcomes	Main Findings	Limitations
Linassi et al. [21], 2025	Prospective, single-center, observational study	14 adult females undergoing breast cancer surgery	Propofol TCI (Eleveld model; median effect-site CeP 2.75 µg/mL [1.92–3.48]); remifentanil TCI (Minto model; median effect-site CeR 3 ng/mL [3–3])	Single ketamine bolus targeting effect-site concentration CeK 1 µg/mL (Domino model; median dose 0.57 mg/kg)	BIS and SEF95 showed CeK-dependent biphasic changes; SPI unchanged	Peak BIS/SEF95 occurred at lower CeK (0.2–0.5 µg/mL) several minutes post-bolus; clinical BIS may be misleading	No control; female only; no ketamine plasma levels measured; DSA was generated and analyzed but not included in the outcomes
Araujo et al. [22], 2017	Prospective, single-blind, randomized controlled study	39 adults undergoing elective spine surgery	Propofol at 200 mL/h until LOC via TCI; remifentanil TCI at effect-site CeR 3 ng/mL; rocuronium 0.6 mg/kg	Randomized ketamine boluses: 0.2, 0.5, or 1 mg/kg	BIS, SEF, and DSA recorded during 9 min baseline and 9 min post-ketamine; ketamine increased both BIS and SEF dose-dependently	Dose-related increases in BIS and SEF; DSA showed shift toward higher frequency power; BIS/SEF increase not reflecting lighter hypnosis	Single-blind; no surgical stimulus during observation; age differences between groups
Tanaka et al. [23] 2025	Prospective, observational	19 who underwent abdominal, gynaecological, and breast surgeries	Propofol TCI 3–5 mcg/mL; remifentanil 0.07–0.33 mcg/kg/min); rocuronium (no dose reported)	After a stable propofol infusion for at least 10 min, ketamine was administered as a 0.5 mg/kg bolus, followed by a continuous infusion at 0.125 mg/kg/h	BIS, SEF95, DSA, phase coupling, and modulation index	Low-dose ketamine increased BIS and SEF95; DSA revealed a decrease in alpha and delta power and an increase in beta power after low-dose ketamine	Non-blinded; female only; no power calculation; no control group; missing information on TCI models; missing information on dose of remifentanil and propofol during ketamine bolus and infusion

**Table 2:** Risk Of Bias In Non-randomized Studies of Interventions (ROBINS-I) tool summary for Linassi et al. [21].

Domain	Judgment
D1—Confounding	🔴 Serious
D2—Selection of participants	🔴 Serious
D3—Classification of interventions	🟡 Moderate
D4—Deviations from intended interventions	🟢 Low
D5—Missing outcome data	🟢 Low
D6—Measurement of outcomes	🟡 Moderate
D7—Selection of reported result	🟡 Low–Moderate
**Overall**	🔴 **Serious**

**Table 3:** The Cochrane Risk of Bias 2 (RoB 2) tool for Aroujo et al. [22].

Domain	Judgment
D1—R1	🟡 Some Concerns
D2—Deviations	🟢 Low
D3—Missing data	🟢 Low
D4—Outcome measurement	🟡 Some Concerns
D5—Reported result	🟡 Some Concerns
**Overall**	🟡 **Some Concerns**

**Table 4:** Risk Of Bias In Non-randomized Studies of Interventions (ROBINS-I) tool summary for Tanaka et al. [23].

Domain	Judgment
D1—Confounding	🟡 Moderate
D2—Selection of participants	🟢 Low
D3—Classification of interventions	🟢 Low
D4—Deviations from intended interventions	🟡 Moderate
D5—Missing outcome data	🟢 Low
D6—Measurement of outcomes	🟢 Low
D7—Selection of reported result	🟢 Low
**Overall**	**Moderate**

## Data Availability

Data will be available upon reasonable request to the corresponding author.

## Supplementary Materials

The following supporting information can be downloaded at: https://www.mdpi.com/article/10.3390/jpm16080406/s1, Table S1: PRISMA 2020 for Abstracts Checklist; Table S2: PRISMA 2020 Checklist; Table S3: Full-text studies excluded from the systematic review and reasons for exclusion; Supplementary File S1: Complete search strategy. The same combination of keywords was used for PubMed, Embase, and the Cochrane Library. No additional datasets, data extraction forms, or analytic code were generated or are publicly available.

## Abbreviations

The following abbreviations are used in this manuscript:

GA	General anesthesia
EEG	Electroencephalographic
DSA	Density spectral array
DOA	Depth of anesthesia
ESAIC	European Society of Anaesthesiology and Intensive Care
MAC	Minimum Alveolar Concentration
TCI	Target-controlled infusion
PK/PD	Pharmacokinetic/pharmacodynamic
NONMEM	Nonlinear mixed-effects models
NMDA	N-methyl-D-aspartate
BIS	Bispectral Index
SEF	Spectral Edge Frequency
SPI	Surgical Pleth Index
TCI-TIVA	Target-controlled infusion total intravenous anesthesia
