# Supplementary material for: Electroencephalographic Patterns Associated with the Pharmacokinetics and Pharmacodynamics of GABAergic Agents, Opioids, and Ketamine During General Anesthesia: A Systematic Review
Source: J Pers Med. 2026 Jul 29;16(8):406. doi: 10.3390/jpm16080406 (PMC13514481; doi:10.3390/jpm16080406)
Supplement: Supplementary file 1 [file jpm-16-00406-s001.zip › jpm-4409843-supplementary.pdf]

Supplementary Table S1. PRISMA 2020 for Abstracts Checklist

Section and Topic	Item #	Checklist item	Reported (Yes/No)
**TITLE**			
Title	1	Identify the report as a systematic review.	Yes
**BACKGROUND**			
Objectives	2	Provide an explicit statement of the main objective(s) or question(s) the review addresses.	Yes
**METHODS**			
Eligibility criteria	3	Specify the inclusion and exclusion criteria for the review.	Yes
Information sources	4	Specify the information sources (e.g. databases, registers) used to identify studies and the date when each was last searched.	Yes
Risk of bias	5	Specify the methods used to assess risk of bias in the included studies.	Yes
Synthesis of results	6	Specify the methods used to present and synthesise results.	Yes
**RESULTS**			
Included studies	7	Give the total number of included studies and participants and summarise relevant characteristics of studies.	Yes
Synthesis of results	8	Present results for main outcomes, preferably indicating the number of included studies and participants for each. If meta-analysis was done, report the summary estimate and confidence/credible interval. If comparing groups, indicate the direction of the effect (i.e. which group is favoured).	Yes
**DISCUSSION**			
Limitations of evidence	9	Provide a brief summary of the limitations of the evidence included in the review (e.g. study risk of bias, inconsistency and imprecision).	Yes
Interpretation	10	Provide a general interpretation of the results and important implications.	Yes
**OTHER**			
Funding	11	Specify the primary source of funding for the review.	Yes
Registration	12	Provide the register name and registration number.	Yes

From: Page MJ, McKenzie JE, Bossuyt PM, Boutron I, Hoffmann TC, Mulrow CD, et al. The PRISMA 2020 statement: an updated guideline for reporting systematic reviews. BMJ 2021;372:n71. doi: 10.1136/bmj.n71. This work is licensed under CC BY 4.0. To view a copy of this license, visit https://creativecommons.org/licenses/by/4.0/

Supplementary Table S2. PRISMA 2020 Checklist.

Section and Topic	Item #	Checklist item	Location where item is reported
**TITLE**			
Title	1	Identify the report as a systematic review.	Title
**ABSTRACT**			
Abstract	2	See the PRISMA 2020 for Abstracts checklist.	Abstract
**INTRODUCTION**			
Rationale	3	Describe the rationale for the review in the context of existing knowledge.	Introduction, Page 3
Objectives	4	Provide an explicit statement of the objective(s) or question(s) the review addresses.	Introduction, Page 3
**METHODS**			
Eligibility criteria	5	Specify the inclusion and exclusion criteria for the review and how studies were grouped for the syntheses.	Section 2, Methods, Pages 5-6
Information sources	6	Specify all databases, registers, websites, organisations, reference lists and other sources searched or consulted to identify studies. Specify the date when each source was last searched or consulted.	Section 2, Methods, Pages 5-6
Search strategy	7	Present the full search strategies for all databases, registers and websites, including any filters and limits used.	Section 2, Methods, Pages 5-6
Selection process	8	Specify the methods used to decide whether a study met the inclusion criteria of the review, including how many reviewers screened each record and each report retrieved, whether they worked independently, and if applicable, details of automation tools used in the process.	Section 2, Methods, Pages 5-6
Data collection process	9	Specify the methods used to collect data from reports, including how many reviewers collected data from each report, whether they worked independently, any processes for obtaining or confirming data from study investigators, and if applicable, details of automation tools used in the process.	Section 2, Methods, Pages 5-6
Data items	10a	List and define all outcomes for which data were sought. Specify whether all results that were compatible with each outcome domain in each study were sought (e.g. for all measures, time points, analyses), and if not, the methods used to decide which results to collect.	Section 2, Methods, Pages 5-6
	10b	List and define all other variables for which data were sought (e.g. participant and intervention characteristics, funding sources). Describe any assumptions made about any missing or unclear information.	Section 2, Methods, Pages 5-6
Study risk of bias assessment	11	Specify the methods used to assess risk of bias in the included studies, including details of the tool(s) used, how many reviewers assessed each study and whether they worked independently, and if applicable, details of automation tools used in the process.	Section 2, Methods, Pages 5-6
Effect measures	12	Specify for each outcome the effect measure(s) (e.g. risk ratio, mean difference) used in the synthesis or presentation of results.	Not applicable

Section and Topic	Item #	Checklist item	Location where item is reported
Synthesis methods	13a	Describe the processes used to decide which studies were eligible for each synthesis (e.g. tabulating the study intervention characteristics and comparing against the planned groups for each synthesis (item #5)).	Section 2, Methods, Pages 5-6
	13b	Describe any methods required to prepare the data for presentation or synthesis, such as handling of missing summary statistics, or data conversions.	Not applicable
	13c	Describe any methods used to tabulate or visually display results of individual studies and syntheses.	Table1; (Page 6) Figure 4 (page 7)
	13d	Describe any methods used to synthesize results and provide a rationale for the choice(s). If meta-analysis was performed, describe the model(s), method(s) to identify the presence and extent of statistical heterogeneity, and software package(s) used.	Section 2, Methods, Pages 5-6 (narrative synthesis stated; meta-analysis not feasible)
	13e	Describe any methods used to explore possible causes of heterogeneity among study results (e.g. subgroup analysis, meta-regression).	Not applicable
	13f	Describe any sensitivity analyses conducted to assess robustness of the synthesized results.	Not applicable
Reporting bias assessment	14	Describe any methods used to assess risk of bias due to missing results in a synthesis (arising from reporting biases).	Not applicable
Certainty assessment	15	Describe any methods used to assess certainty (or confidence) in the body of evidence for an outcome.	Section 2, Methods, Pages 5-6
**RESULTS**			
Study selection	16a	Describe the results of the search and selection process, from the number of records identified in the search to the number of studies included in the review, ideally using a flow diagram.	Section 3, Results; (Pages 8-10) Figure 4 (page 7)
	16b	Cite studies that might appear to meet the inclusion criteria, but which were excluded, and explain why they were excluded.	Figure 4 (page 7)
Study characteristics	17	Cite each included study and present its characteristics.	Table 1 (Page 6)
Risk of bias in studies	18	Present assessments of risk of bias for each included study.	Section 3, Results

Section and Topic	Item #	Checklist item	Location where item is reported
			(Pages 8-10)
Results of individual studies	19	For all outcomes, present, for each study: (a) summary statistics for each group (where appropriate) and (b) an effect estimate and its precision (e.g. confidence/credible interval), ideally using structured tables or plots.	Section 3, Results (Pages 8-10)
Results of syntheses	20a	For each synthesis, briefly summarise the characteristics and risk of bias among contributing studies.	Section 4, Discussion
	20b	Present results of all statistical syntheses conducted. If meta-analysis was done, present for each the summary estimate and its precision (e.g. confidence/credible interval) and measures of statistical heterogeneity. If comparing groups, describe the direction of the effect.	Not applicable (narrative synthesis stated; meta-analysis not feasible)
	20c	Present results of all investigations of possible causes of heterogeneity among study results.	Not applicable
	20d	Present results of all sensitivity analyses conducted to assess the robustness of the synthesized results.	Not applicable
Reporting biases	21	Present assessments of risk of bias due to missing results (arising from reporting biases) for each synthesis assessed.	Not applicable
Certainty of evidence	22	Present assessments of certainty (or confidence) in the body of evidence for each outcome assessed.	Section 3-4
**DISCUSSION**			
Discussion	23a	Provide a general interpretation of the results in the context of other evidence.	Section 4, Discussion, (Pages 11-14)
	23b	Discuss any limitations of the evidence included in the review.	Section 4, Discussion, (Pages 11-14)
	23c	Discuss any limitations of the review processes used.	Section 4, Discussion, (Pages 11-14)
	23d	Discuss implications of the results for practice, policy, and future research.	Section 5, Conclusion, (Page 15)
**OTHER INFORMATION**			
Registration and protocol	24a	Provide registration information for the review, including register name and registration number, or state that the review was not registered.	Abstract
	24b	Indicate where the review protocol can be accessed, or state that a protocol was not prepared.	Section 2, Methods,

Section and Topic	Item #	Checklist item	Location where item is reported
			(Page 5)
	24c	Describe and explain any amendments to information provided at registration or in the protocol.	Section 2, Methods,
Support	25	Describe sources of financial or non-financial support for the review, and the role of the funders or sponsors in the review.	Abstract
Competing interests	26	Declare any competing interests of review authors.	Page 15
Availability of data, code and other materials	27	Report which of the following are publicly available and where they can be found: template data collection forms; data extracted from included studies; data used for all analyses; analytic code; any other materials used in the review.	Page 15

From: Page MJ, McKenzie JE, Bossuyt PM, Boutron I, Hoffmann TC, Mulrow CD, et al. The PRISMA 2020 statement: an updated guideline for reporting systematic reviews. BMJ 2021;372:n71. doi: 10.1136/bmj.n71. This work is licensed under CC BY 4.0. To view a copy of this license, visit https://creativecommons.org/licenses/by/4.0/

Supplementary Table S3. Full-text studies excluded from the systematic review and reasons for exclusion.

Reference	Reason for exclusion
**Wrong outcome**	
Schüler, S.S.; Petersen, C.L.; West, N.C.; et al. *The effect of ketamine on depth of hypnosis indices during total intravenous anesthesia—a comparative study using a novel electroencephalography case replay system.* **J. Clin. Monit. Comput. 2021**, *35*, 1027–1036. https://doi.org/10.1007/s10877-020-00565-0	Did not report raw EEG data or density spectral array (DSA) patterns.
Matsushita, S.; Oda, S.; Otaki, K.; et al. *Change in auditory evoked potential index and bispectral index during induction of anesthesia with anesthetic drugs.* **J. Clin. Monit. Comput. 2015**, *29*, 621–626. https://doi.org/10.1007/s10877-014-9643-x	Did not report raw EEG data or DSA patterns.
Hasanein, R.; El-Sayed, W.; Nabil, N.; Elsayed, G. *The effect of combined remifentanil and low dose ketamine infusion in patients undergoing laparoscopic gastric bypass.* **Egypt. J. Anaesth. 2011**, *27*, 255–260. https://doi.org/10.1016/j.egja.2011.07.009	Did not report EEG/DSA patterns relevant to the review objective.
Faraoni, D.; Salengros, J.C.; Engelman, E.; Ickx, B.; Barvais, L. *Ketamine has no effect on bispectral index during stable propofol–remifentanil anaesthesia.* **Br. J. Anaesth. 2009**, *102*, 336–339. https://doi.org/10.1093/bja/aen403	Reported processed EEG indices only, without raw EEG or DSA patterns.
Schueler, S.; Petersen, C.L.; Ansermino, J.M.; Merchant, R.; Gorges, M. *The effect of ketamine on depth of hypnosis indices during total intravenous anesthesia—a comparative study using a novel case replay system.* **Anesth. Analg. 2018**, *127*, 107–108. https://doi.org/10.1002/central/CN-01620832	Conference abstract with insufficient raw EEG/DSA data.
**Wrong study design**	
Akhlaghi, N.; Kaviani, N.; Eshghi, A.; Tahririan, D.; Mohammadpour, M. *Comparative evaluation of bispectral index system after sedation with midazolam and propofol combined with remifentanil versus ketamine in uncooperative patients during dental procedures.* **Dent. Res. J. 2016**, *13*, 1–6. https://doi.org/10.4103/1735-3327.174688	Conducted during procedural sedation rather than general anesthesia.
**Wrong language**	
Chaaben, K.; Marret, E.; Lamonerie, L.; Lembert, N.; Bonnet, F. *La kétamine à dose antihyperalgésique augmente l'index bispectral.* **Ann. Fr. Anesth. Réanim. 2004**, *23*, 513–516. https://doi.org/10.1016/j.annfar.2004.02.048	Published in French (non-English article).

Supplementary File S1. Complete search strategy. The same combination of keywords was used for PubMed, Embase, and the Cochrane Library. The search strategy was based on the following terms:

(((propofol OR GABAergic) AND (remifentanil OR opioid)) AND ketamine)
AND
(
EEG
OR electroencephalography
OR BIS
OR "bispectral index"
OR SEF95
OR "spectral edge frequency"
OR PSI
OR "Patient State Index"
OR neuromonitoring
OR "theta wave"
OR "beta wave"
OR "depth of anesthesia monitoring"
OR "Intraoperative Neurophysiological Monitoring"[Mesh]
)
